# A dangerous loop

**DOI:** 10.1002/ccr3.563

**Published:** 2016-04-15

**Authors:** Saqib Ahmad, Damian Bragg, Abed M. Zaitoun, Dileep N. Lobo

**Affiliations:** ^1^ GastroenterologyNational Institute for Health Research Nottingham Digestive Diseases Biomedical Research UnitNottingham University Hospitals and University of NottinghamQueen's Medical CentreNottinghamUK; ^2^ Gastrointestinal SurgeryNational Institute for Health Research Nottingham Digestive Diseases Biomedical Research UnitNottingham University Hospitals and University of NottinghamQueen's Medical CentreNottinghamUK; ^3^ Cellular PathologyNational Institute for Health Research Nottingham Digestive Diseases Biomedical Research UnitNottingham University Hospitals and University of NottinghamQueen's Medical CentreNottinghamUK

**Keywords:** Adverse event, ERCP, hemorrhage, imaging, splenic injury, surgery

## Abstract

A 76‐year‐old man developed a hemoperitoneum after ERCP for choledocholithiasis. He underwent a laparotomy and splenectomy for a capsular tear at the splenic hilum, a rare complication of ERCP. “Bowing” of the endoscope with torsion on the greater curvature of the stomach may lead to shear forces causing splenic injury.

## Scenario

A 76‐year‐old man underwent ERCP for choledocholithiasis. Intubation and ampullary cannulation were performed without difficulty. A cholangiogram revealed two common bile duct stones, which were extracted after endoscopic sphincterotomy and a balloon trawl.

**Figure 1:**
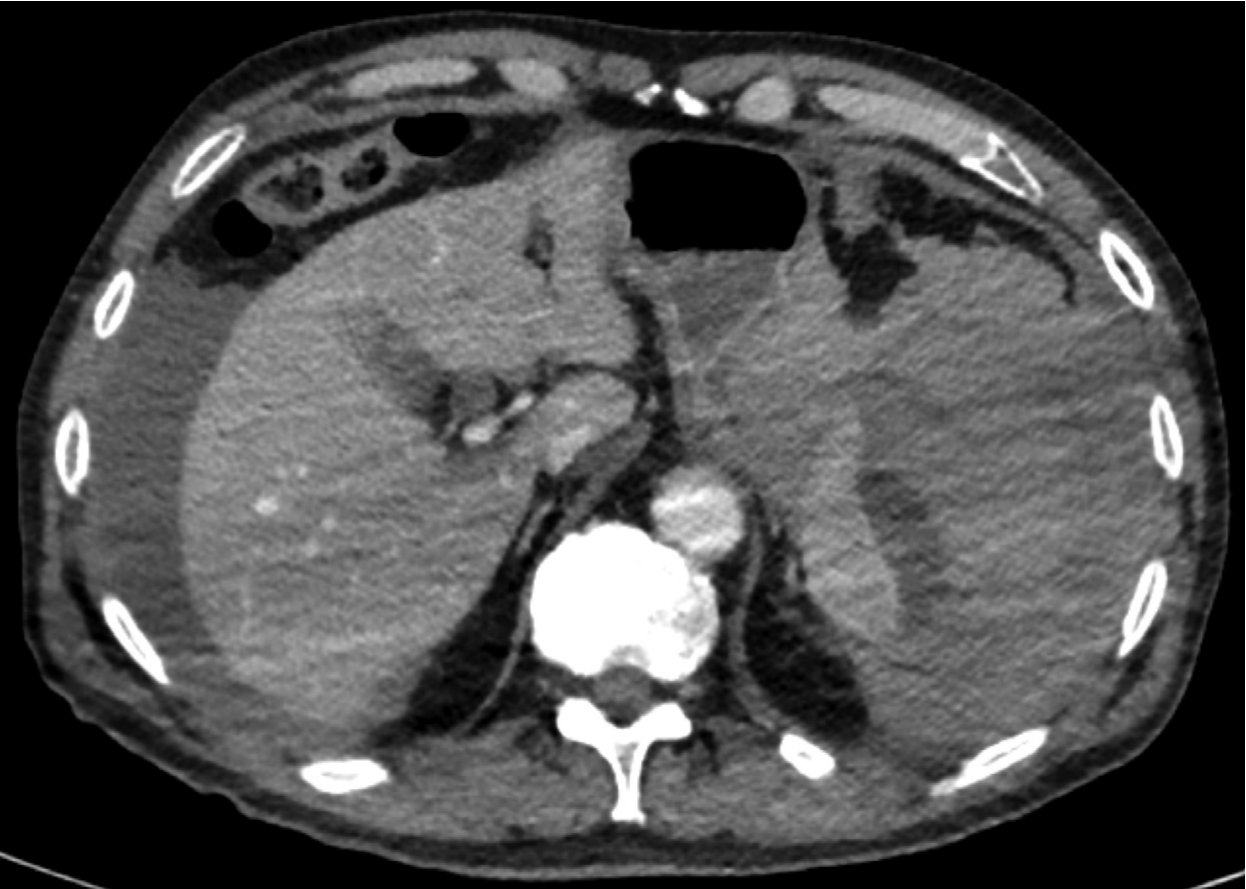
A postresuscitation abdominal CT scan image.

He complained of abdominal pain 30 min after the procedure. He was tachycardic (98 bpm) and hypotensive (80/40 mm Hg), and had a tender epigastrium. His hemoglobin dropped from 138 to 85 g/L. A postresuscitation CT scan image is shown in Figure 1.

## Question

What is the diagnosis?

## Answer

**Figure 2:**
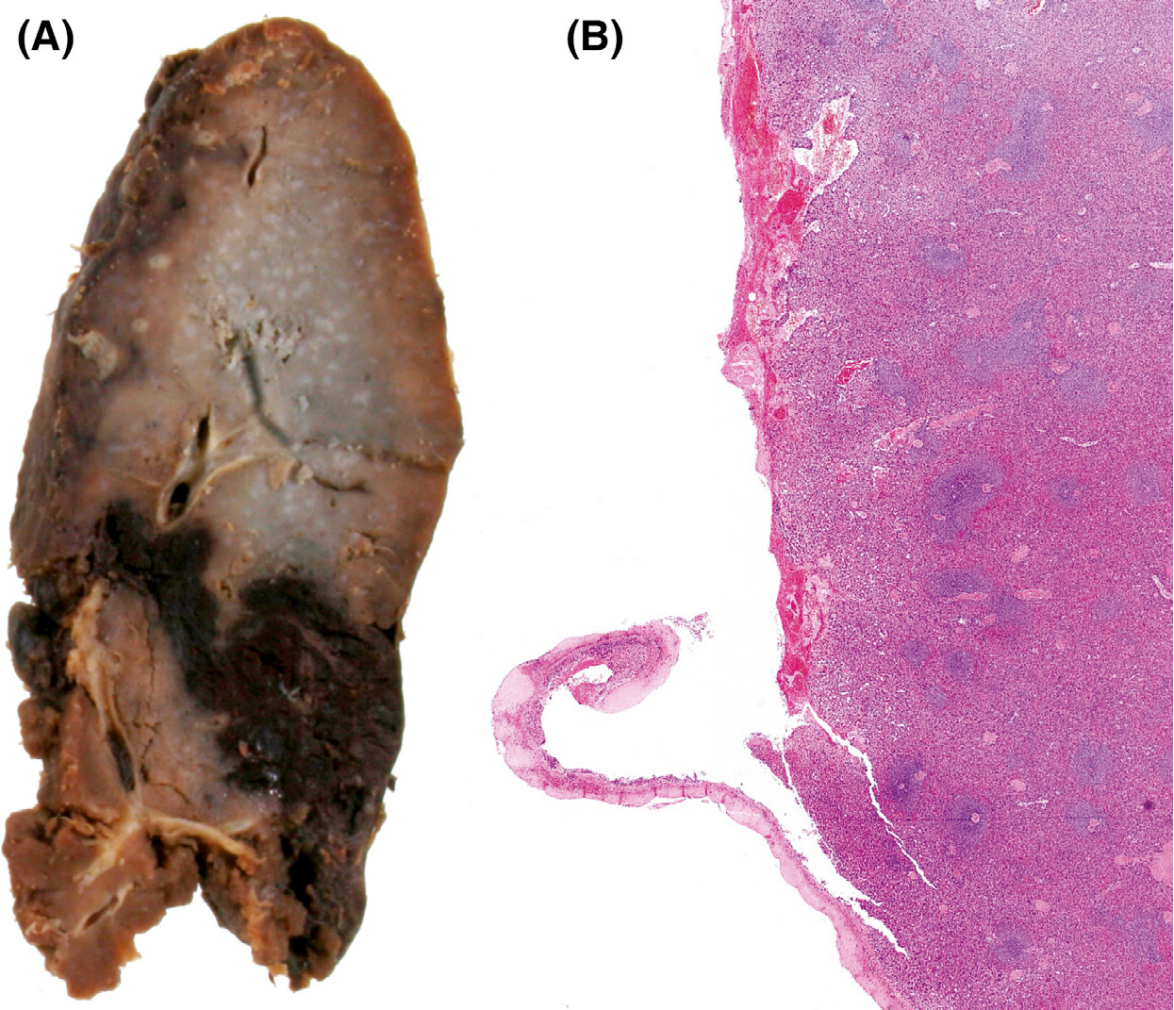
Pathological examination showing a normal‐sized spleen with a capsular tear on the hilar surface and a subcapsular hematoma (A). This was confirmed on histology (B).

The CT scan shows a hemoperitoneum. Large amounts of intraperitoneal blood and clots were found at emergency laparotomy along with fresh bleeding from a capsular tear in the splenic hilum. He had a splenectomy, from which he recovered. Pathological examination showed a normal‐sized spleen with a capsular tear on the hilar surface and a subcapsular hematoma (Fig. 2A), confirmed on histology (Fig. 2B).

Although splenic injury is a well‐recognized adverse event of colonoscopy, only 12 cases after ERCP have been reported previously. The likely mechanism is that “bowing” of the endoscope in the long position with torsion on the greater curvature of the stomach may lead to shear forces, resulting in splenic vascular avulsion or capsular tears. Overinflation of the stomach, difficulty in duodenal intubation, and prolonged procedure time, leading to excessive scope manipulation and torsion on the shaft, are also proposed as risk factors for splenic injury during ERCP and upper gastrointestinal endoscopy 1, 2. Gentle manual epigastric pressure to help prevent excessive loop formation during difficult duodenal intubation may help prevent splenic injury.

## Conflict of Interest

None declared.
